# Beneficial implications of adjuvant chemotherapy for stage IB lung adenocarcinoma exhibiting elevated SUVmax in FDG-PET/CT: a retrospective study from a single center

**DOI:** 10.3389/fonc.2024.1367200

**Published:** 2024-03-11

**Authors:** Miao Huang, Bing Liu, Xiang Li, Nan Li, Xin Yang, Yaqi Wang, Shanyuan Zhang, Fangliang Lu, Shaolei Li, Shi Yan, Nan Wu

**Affiliations:** ^1^ Key Laboratory of Carcinogenesis and Translational Research (Ministry of Education), Department of Thoracic Surgery II, Peking University Cancer Hospital and Institute, Beijing, China; ^2^ Key Laboratory of Carcinogenesis and Translational Research (Ministry of Education), Department of Nuclear Medicine, Peking University Cancer Hospital and Institute, Beijing, China; ^3^ Key Laboratory of Carcinogenesis and Translational Research (Ministry of Education), Department of Pathology, Peking University Cancer Hospital and Institute, Beijing, China; ^4^ State Key Laboratory of Molecular Oncology, Department of Thoracic Surgery II, Peking University Cancer Hospital and Institute, Beijing, China

**Keywords:** adjuvant chemotherapy, lung adenocarcinoma, maximum standardized uptake value, standardized uptake value, overall survival

## Abstract

**Background:**

Controversy surrounds the efficacy of adjuvant chemotherapy (ACT) in the treatment of stage I lung adenocarcinoma (LUAD). The objective of this study was to examine the impact of the maximum standardized uptake value (SUVmax) as measured by 18F-fluorodeoxyglucose positron emission tomography/computed tomography (FDG-PET/CT) on the efficacy of ACT in patients diagnosed with stage I LUAD.

**Methods:**

We scrutinized the medical records of 928 consecutive patients who underwent complete surgical resection for pathological stage I LUAD at our institution. The ideal cut-off value for primary tumor SUVmax in terms of disease-free survival (DFS) and overall survival (OS) was determined using the X-tile software. The Kaplan–Meier method and Cox regression analysis were used for survival analysis.

**Results:**

Based on the SUVmax algorithm, the ideal cutoff values were determined to be 4.9 for DFS and 5.0 for OS. We selected 5.0 as the threshold because OS is the more widely accepted predictive endpoint. In a multivariate Cox regression analysis, SUVmax ≥ 5.0, problematic IB stage, and sublobectomy were identified as independent risk factors for poor DFS and OS. It is noteworthy that patients who were administered ACT had significantly longer DFS and OS than what was observed in the subgroup of patients with pathological stage IB LUAD and SUVmax ≥ 5.0 (p < 0.035 and p ≤ 0.046, respectively). However, there was no observed survival advantage for patients in stages IA or IB who had an SUVmax < 5.0.

**Conclusion:**

The preoperative SUVmax of tumors served as an indicator of the impact of ACT in the context of completely resected pathological stage I LUAD. Notably, patients within the Stage IB category exhibiting elevated SUVmax levels emerged as a subgroup experiencing substantial benefits from postoperative ACT.

## Introduction

Non-small cell lung cancer (NSCLC) stands as the foremost cause of cancer-related mortality worldwide, with surgery serving as the primary therapeutic modality for patients in the early stages (1, 2). Despite the implementation of radical resection, roughly half of the patients with NSCLC who are surgically treated, encounter recurrence (3, 4). Recent findings from a comprehensive multi-centric observational study indicate 5-year overall survival (OS) rates of 93.2% and 82.7% for patients in pathological stages IA and IB (8^th^ edition), respectively (5). Postoperative ACT plays a crucial role in enhancing prognosis, with reported 5-year OS benefits ranging from 5% to 10% through cisplatin-based ACT in numerous large randomized clinical trials and meta-analyses (6–10). While ACT is recommended for patients with resected stage II and IIIA NSCLC, its application in stage I patients remains a subject of debate (11, 12). Various retrospective studies have demonstrated diverse degrees of survival advantage with ACT in stage I patients (13–16). However, the lack of robust evidence has precluded the endorsement of ACT for stage I patients in the current National Comprehensive Cancer Network (NCCN) guidelines, except for select stage IB patients exhibiting high-risk factors (17). The limited efficacy of ACT in stage I NSCLCs may be attributed, in part, to the inclusion of a substantial number of patients with favorable prognoses who may not require adjuvant treatment. Consequently, the identification of patients at high risk of recurrence is imperative for discerning the potential benefits of ACT.

LUAD constitutes the predominant histological subtype of NSCLC, representing approximately 50% of surgically resected tumors (18). Despite sharing the same TNM stage, LUAD exhibits considerable histological and molecular heterogeneity, influencing prognosis and treatment decisions (19). Therefore, additional predictive factors are essential for identifying patients diagnosed with stage I LUAD at an elevated risk of recurrence who would derive greater benefits from ACT. Among preoperative variables, the SUVmax derived from 18F-fluorodeoxyglucose (FDG) PET/CT scans stands out as a superior predictor of tumor invasiveness and prognosis (20–23). Platinum-doublet regimens represent a widely employed chemotherapeutic approach in LUAD adjuvant treatment, despite their associated high toxicity (24). The efficacy of platinum-based chemotherapy is intricately linked to the metabolic activity of lung cancer, a parameter encapsulated by SUVmax on FDG-PET/CT (25). However, the extent to which the SUVmax of the primary tumor can prognosticate the effectiveness of ACT following surgery in early-stage LUAD remains uncertain.

To further assess the value of SUVmax in predicting the outcomes of ACT in patients with pathological stage I LUAD, we conducted a retrospective analysis, using the database of our institute.

## Materials and methods

### Patients

In this retrospective analysis, we systematically examined the electronic medical records of patients diagnosed with early-stage NSCLC who had undergone surgical resection at the Department of Thoracic Surgery II, Peking University Cancer Hospital. All enrolled patients underwent preoperative staging and surgical interventions in accordance with the guidelines outlined by the NCCN for lung cancer (version 2012). Preoperative staging procedures included chest CT, brain MRI, abdominal ultrasonography, bone scintigraphy, and, when feasible, whole-body ^18^F-FDG PET/CT scans. Anatomic pulmonary resection with systematic mediastinal lymphadenectomy, involving the dissection of at least three N2 stations, was scheduled for all patients. Sublobectomy (segmentectomy or wedge resection) with systematic lymph node sampling was selectively performed in specific cases. To ensure precise N staging, intrapulmonary lymph nodes (stations 13, 14) were meticulously removed from resected specimens by surgeons and subjected to pathological review.

Patients diagnosed with pathological stage I invasive LUAD who had undergone PET/CT scans prior to surgery were included. Exclusion criteria encompassed individuals meeting one or more of the following conditions: (1) multiple primary lung cancers, (2) concurrent malignancies within the preceding 5 years, (3) received neoadjuvant therapy or any other form of adjuvant therapy (including targeted therapy or radiotherapy), (4) R1/R2 resection, and (5) inadequate follow-up information or mortality within 90 days post-surgery. Clinical data pertaining to the enrolled patients were meticulously retrieved from our prospectively maintained database. Ethical considerations were adhered to in accordance with the Declaration of Helsinki, and approval was obtained from the Ethics Committee of Peking University Cancer Hospital and Institute (Institutional Review Board No. 2019KT59). Given the retrospective nature of the study, the necessity for informed consent was waived.

### Integrated PET/CT imaging

Preoperative PET/CT examinations were conducted using a Gemini TF PET/CT system manufactured by Philips. Prior to the procedure, patients observed a minimum fasting period of 6 hours and underwent a 60-minute resting period. Subsequently, an intravenous injection of 3.7 MBq of 18FDG/kg of body weight was administered. Patients assumed a supine position during PET/CT data acquisition. Emission images were obtained subsequent to CT scanning, and the emission scan comprised 8 to 10 bed positions, each lasting 1 minute per step. The FDG uptake of the tumor was visually assessed in comparison to the surrounding tissue in regions devoid of significant artifacts and overlapping increased FDG uptake organs. Independent evaluation of integrated PET/CT images was conducted by a team of experienced radiologists. The SUVmax of the primary tumor was meticulously recorded. All integrated PET/CT imaging procedures were conducted within the 4-week interval preceding the scheduled surgery.

### Histopathologic evaluation

Resected specimens underwent standard pathological analysis and were histologically examined by experienced pulmonary pathologists. Routine records included crucial pathological features such as the degree of differentiation, tumor size, lympho vascular invasion (LVI), and visceral pleural invasion (VPI). Tumors were deemed LVI-positive when cancer cells were identified within the intra tumoral lumen, comprising vessels or lymphatics, given the inherent difficulty in precisely distinguishing between vascular and lymphatic invasion. Elastic-van-Gieson staining was performed when tumors abutted the visceral pleura or exhibited pleural puckering. The American Joint Committee on Cancer (AJCC) Cancer Staging Manual, 8^th^ edition, served as the basis for pathological staging.

### Adjuvant chemotherapy

Adjuvant chemotherapy was administered to patients with high-risk factors, encompassing poorly differentiated tumors, VPI (+), LVI (+), and sublobectomy (26). The treatment protocol comprised four cycles of platinum doublets (either cisplatin in combination with paclitaxel or pemetrexed), with a three-week interval between cycles. Patient eligibility for platinum-based ACT was determined based on age and performance status, with the informed consent of the patient. Initiation of ACT occurred within a window of 4 to 8 weeks following surgical resection. Adverse events associated with chemotherapy were systematically documented, and decisions regarding dose adjustments or treatment delays were made by seasoned oncologists prior to scheduled administrations, considering both objective criteria (white cell count, absolute neutrophil count, serum creatinine, gastrointestinal symptoms, and neurologic symptoms) and subjective criteria (performance status) (27). Notably, no instances of mortality attributed to chemotherapy were observed among patients completing the stipulated four cycles of ACT.

### Postoperative follow-up

All the patients underwent a structured follow-up regimen, with assessments conducted at three-month intervals during the initial two years, followed by semi-annual evaluations over the subsequent three years, and subsequently transitioning to annual examinations. The follow-up protocol included chest CT, abdominal and cervical lymph node ultrasound examinations, and serum tumor marker assessments every three months. Additionally, an annual MRI of the brain was performed for each participant. In cases where patients were not available for follow-up at our institution, survival data were obtained through telephone communication. Locoregional recurrence or distant metastasis was ascertained through comprehensive examinations, encompassing chest CT, abdominal ultrasound, brain MRI, and bone scintigraphy. A PET-CT scan was recommended if necessary. DFS was operationally defined as the duration from the date of surgery to the occurrence of disease recurrence, death, or the last follow-up. OS was defined as the interval from the date of surgery to death or the last follow-up. The concluding follow-up assessment was conducted in June 2023.

### Statistical analysis

The X-tile software was used to establish the cutoff value for SUVmax, which was determined by identifying the value associated with the minimum P-values derived from log-rank chi-squared statistics for the categorical SUVmax with respect to DFS and OS (28). Associations between clinicopathological characteristics were scrutinized through the application of the Pearson’s chi-squared test or Fisher’s exact test for categorical variables. DFS and OS were computed using Kaplan–Meier curves and subjected to comparison using the log-rank test. A multivariate Cox proportional hazards regression model was used to discern independent prognostic factors. All p-values were derived from 2-tailed statistical analyses, and significance was established at p < 0.05. The statistical analysis was conducted using the SPSS software package (version 22.0; SPSS, Chicago, IL, USA).

## Results

### The optimal cut-off value of SUVmax

**Figure 1:**
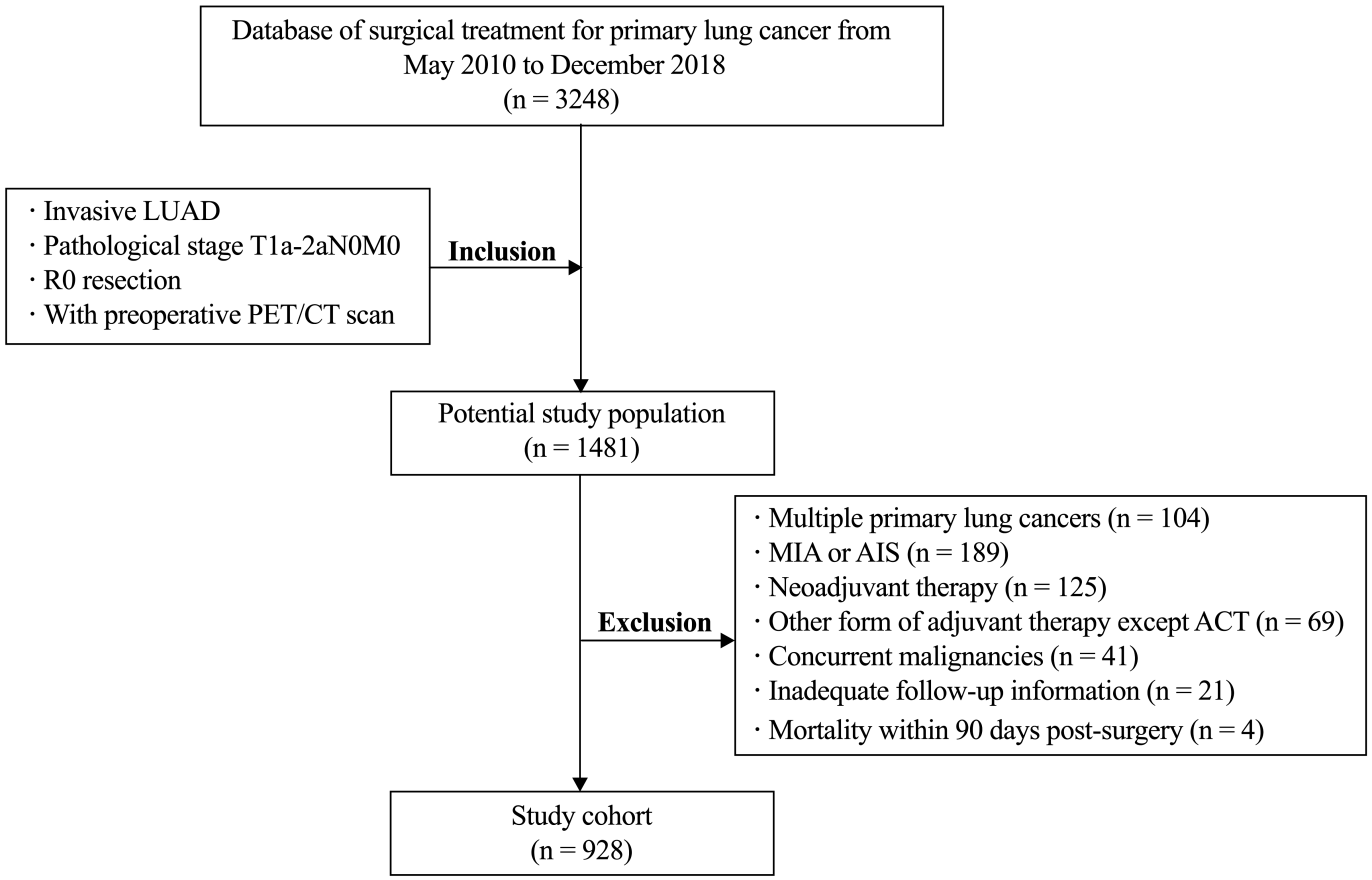
Flow chart of patient selection. LUAD, lung adenocarcinoma; MIA minimally invasive adenocarcinoma; AIS, adenocarcinoma *in situ*; ACT, adjuvant chemotherapy.

A total of 928 consecutive patients diagnosed with pathologically confirmed T1-2aN0M0 invasive LUAD from May 2010 to December 2018 were included in this study, as shown in **Figure 1**. All enrolled patients underwent radical resection, with 827 patients (89.1%) undergoing lobectomy. The median follow-up period was 53.1 months, ranging from 4.7–147.6 months. Among the participants, 81 (8.7%) received postoperative ACT. Ultimately, 116 patients (12.5%) experienced postoperative recurrence, and 60 patients (6.5%) succumbed to lung cancer.

**Figure 2:**
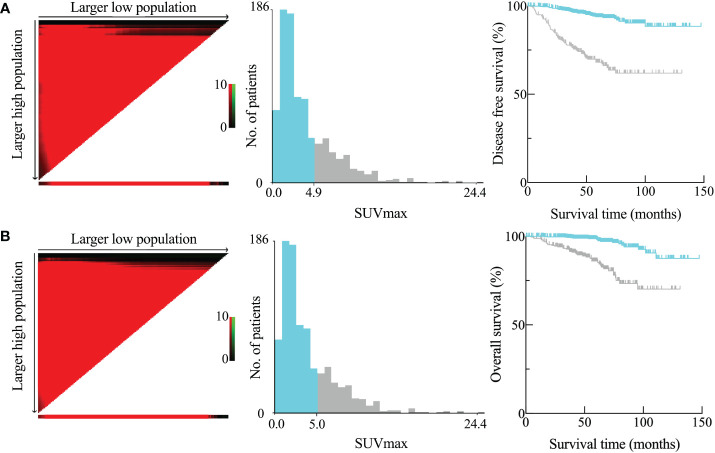
Disease-free survival (DFS) **(A)** and overall survival (OS) **(B)** as determined by the SUVmax of the main tumor using X-tile analysis. SUVmax optimum cutoff values computed for DFS and OS were 4.9 and 5.0, respectively.

The optimal cutoff value for SUVmax to predict DFS and OS was determined using X-tile software. Patient information for DFS and OS, along with the SUVmax of the primary tumor, was input into the X-tile software. The data was analyzed to identify the optimal cutoff value for SUVmax by seeking the maximum difference in survival. The calculated optimal cutoff values were 4.9 for DFS and 5.0 for OS. The corresponding Kaplan-Meier curves, depicted in **Figure 2**, illustrated that a high SUVmax was associated with poorer DFS and OS outcomes.

### Clinicopathological factors associated with high SUVmax

Considering the appropriate SUVmax cutoff value for DFS and OS, we categorized the patients into two groups using the practical threshold of 5.0: “SUVmax < 5.0” and “SUVmax ≥ 5.0.” **Table 1** presents the clinicopathological characteristics of the two groups. Male (p < 0.001), smoking history (p < 0.001), solid nodule (p ≥ 0.001), CEA ≥ 5.0 ng/mL (p < 0.001), tumor size ≥ 3 cm (p < 0.001), VPI+ (p < 0.001), LVI+ (p < 0.001), and pathological T stage (p < 0.001) were significantly correlated with an SUVmax ≥ 5.0. Despite the fact that a greater proportion of patients in the high SUVmax group had undergone lobectomy (p = 0.025) and ACT (p < 0.001), they also had a greater likelihood of lung cancer recurrence (p < 0.001) and lung cancer-related death (p < 0.001).

**Table 1:** Characteristics of patients with low SUVmax (< 5) and high SUVmax (≥ 5).

Variable		SUVmax < 5	SUVmax ≥ 5	*p*-value
		n (%)	n (%)	
Age (years)				0.307
	< 65	464 (69.5)	171 (65.8)	
	≥ 65	204 (30.5)	89 (34.2)	
Gender				< 0.001
	Female	436 (65.3)	130 (50.0)	
	Male	232 (34.7)	130 (50.0)	
Smoking history				< 0.001
	No	498 (74.6)	153 (58.8)	
	Yes	170 (25.4)	107 (41.2)	
Nodule				< 0.001
	Subsolid	341 (51.0)	21 (8.1)	
	Solid	327 (49.0)	239 (91.9)	
CEA (ng/ml)				< 0.001
	< 5	571 (85.5)	167 (64.2)	
	≥ 5	97 (14.5)	93 (35.8)	
Tumor size (cm)				< 0.001
	< 3	628 (94.2)	204 (78.5)	
	≥ 3	39 (5.8)	56 (21.5)	
Visceral pleural invasion				< 0.001
	No	522 (78.1)	150 (57.7)	
	Yes	146 (21.9)	110 (42.3)	
Lymphovascular invasion				< 0.001
	No	656 (98.2)	225 (86.5)	
	Yes	12 (1.8)	35 (13.5)	
Precedure				0.025
	Sublobectomy	89 (13.3)	12 (4.6)	
	Lobectomy	579 (86.7)	248 (95.4)	
pT stage				< 0.001
	T1a	100 (15.0)	3 (1.2)	
	T1b	285 (42.7)	55 (21.2)	
	T1c	121 (18.1)	56 (21.5)	
	T2a	162 (24.3)	146 (56.2)	
Adjuvant chemotherapy				< 0.001
	No	635 (95.1)	212 (81.5)	
	Yes	33 (4.9)	48 (18.5)	
Recurrence				< 0.001
	No	630 (94.3)	182 (70.0)	
	Yes	38 (5.7)	78 (30.0)	
Lung cancer-related death				< 0.001
	No	650 (97.3)	218 (83.8)	
	Yes	18 (2.7)	42 (16.2)	

### Clinicopathological factors associated with survival

The connection between clinical and pathologic variables and DFS and OS for the study cohort was assessed using univariate and multivariate survival analysis, as detailed in **Table 2**. A univariate analysis revealed that several patient characteristics were significant predictive factors for DFS: age (p = 0.042), gender (p = 0.033), smoking history (p = 0.004), CEA level (p = 0.001), SUVmax (p < 0.001), VPI (p < 0.001), LVI (p = 0.038), and TNM stage (p < 0.001). All of the following were significant predictive markers for OS: patient age (p = 0.003), smoking history (p = 0.010), CEA level (p = 0.001), SUVmax (p < 0.001), VPI (p < 0.001), and TNM stage (p < 0.001).

**Table 2:** Cox proportional hazards regression analysis on prognostic factors related to survival in the entire cohort (n=928).

Variable	DFS				OS			
	Univariate	Multivariate			Univariate	Multivariate		
	*p*-value	HR	95% CI	*p*-value	*p*-value	HR	95% CI	*p*-value
Age (≥ 65 vs. < 65)	0.042	1.234	0.838-1.817	0.287	0.003	1.641	0.954-2.822	0.073
Gender (Male vs. Female)	0.033	1.062	0.629-1.796	0.821	0.058	1.086	0.529-2.230	0.822
Smoking history (Yes vs. No)	0.004	1.200	0.699-2.058	0.508	0.010	1.412	0.683-2.922	0.352
CEA (≥ 5 vs. < 5 ng/ml)	0.001	1.111	0.738-1.672	0.616	< 0.001	1.496	0.863-2.596	0.152
SUVmax (≥ 5 vs. < 5)	< 0.001	5.370	3.508-8.220	< 0.001	< 0.001	4.275	2.361-7.741	< 0.001
Visceral pleural invasion (Yes vs. No)	< 0.001	1.002	0.552-1.819	0.994	< 0.001	0.986	0.462-2.104	0.971
Lymphovascular invasion (Yes vs. No)	0.038	0.933	0.440-1.977	0.856	0.181	0.324	0.089-1.178	0.087
Precedure (Sublobectomy vs. Lobectomy)	0.085	2.403	1.410-4.095	0.001	0.116	2.337	1.116-4.895	0.024
Adjuvant chemotherapy (Yes vs. No)	0.080	0.777	0.413-1.462	0.435	0.085	1.447	0.642-3.260	0.373
TNM stage (IB vs. IA)	< 0.001	2.177	1.149-4.126	0.017	< 0.001	4.454	1.772-11.192	0.001

Prognostic factors were incorporated in the Cox proportional hazards regression model, which revealed that SUVmax ≥ 5 was an independent predictor of DFS (HR = 5.370, 95% CI: 3.508–8.220, p < 0.001) and OS (HR = 4.275, 95% CI: 2.361–7.741, p < 0.001). However, multivariate analysis revealed that sublobectomy and problematic IB stage were independent risk factors for poorer DFS and OS, as indicated in **Table 2**. In univariate analysis, ACT did not serve as a predictive factor for DFS (p = 0.080) or OS (p = 0.085) for the entire cohort. Furthermore, ACT did not emerge as an independent predictor of favorable survival in patients with stage I LUAD in multivariate analysis (DFS: HR = 0.777, 95% CI: 0.413–1.462, p = 0.435; OS: HR = 1.447, 95% CI: 0.642–3.260, p = 0.373).

### Potential benefits of ACT in stage IB LUAD with high SUVmax

**Figure 3:**
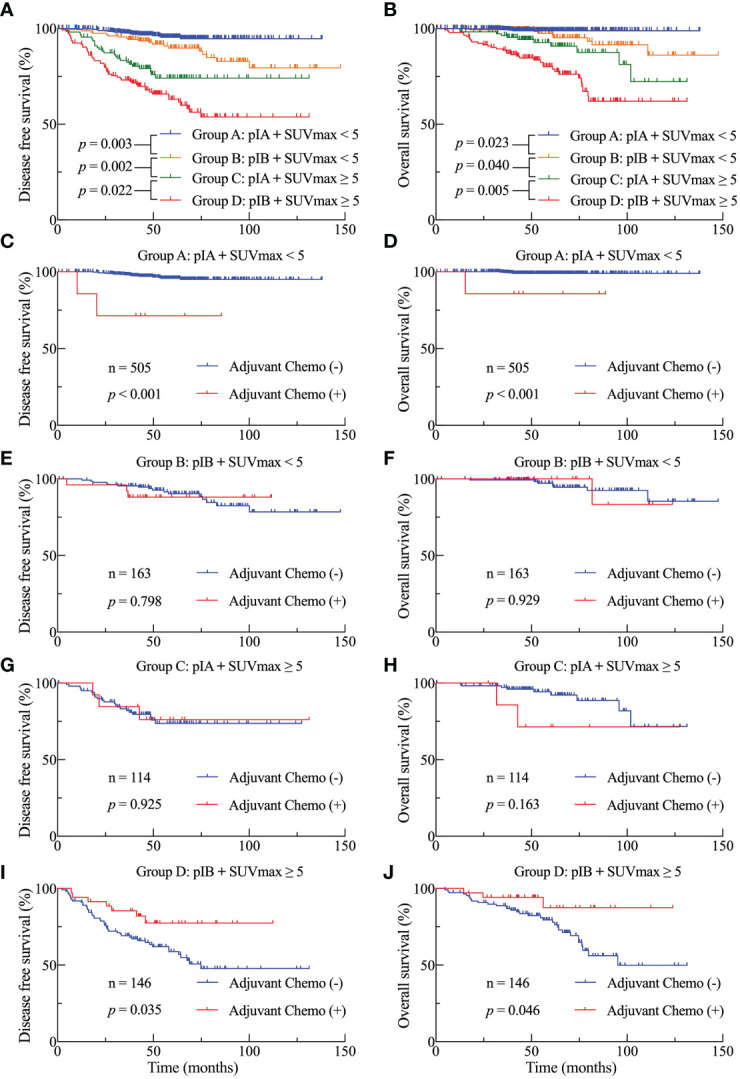
DFS **(A)** and OS **(B)** Kaplan-Meier survival curves subsequent to the division of the cohort into four subgroups based on stage and SUVmax threshold. DFS **(C, E, G, I)** and OS **(D, F, H, J)** curves for the four subgroups in accordance with adjuvant treatment.

Both univariate and multivariate analyses established the primary tumor SUVmax and TNM stage as prognostic factors for survival. Subsequently, we conducted a subgroup analysis to investigate the prognostic significance of SUVmax at various stages. The study cohort was divided into four subgroups based on the SUVmax and TNM stage: group A (pathological stage IA and SUVmax < 5), group B (pathological stage IB and SUVmax < 5), group C (pathological stage IA and SUVmax ≥ 5), and group D (pathological stage IB and SUVmax ≥ 5). As shown in **Figure 3**, both DFS (**Figure 3A**) and OS (**Figure 3B**) among the four subgroups were statistically different. Patients with a greater SUVmax and a more advanced stage exhibited a generally unfavorable prognosis, with notable variations observed within each grouping. Group C exhibited a significantly lower DFS (p = 0.002) and OS (p = 0.040) in comparison to group D.

We investigated the survival benefits of ACT in each subgroup in greater depth. Surprisingly, ACT was linked with poorer OS (**Figure 3D**, p < 0.001) and DFS (**Figure 3C**) among the 505 patients in group A. No statistically significant difference was observed in terms of DFS and OS between patients in groups B and C who had ACT and those who did not (**Figures 3E–H**). Among the 146 patients in group D, patients who were administered ACT had longer DFS (**Figure 3I**, p = 0.035) and OS (**Figure 3J**, p = 0.046) compared to those who were not administered ACT. **Table 3** presents the clinicopathological characteristics of the two subgroups of patients who had received ACT or not in group D patients. Among the predictive factors like CEA, tumor size, VPI and LVI, there was no significant difference between the two subgroups, as shown in **Table 3**. We further analyzed the prognostic factors in the Cox proportional hazards regression model for group D patients. The results revealed that ACT was an independent factor for superior DFS (HR = 0.356, 95% CI: 0.135–0.935, p = 0.036) and OS (HR = 0.334, 95% CI: 0.077–0.852, p = 0.043) in stage IB LUAD patients with high SUVmax (≥ 5), as shown in **Table 4**.

**Table 3:** Characteristics of stage IB patients with high SUVmax (≥ 5).

Variable		Adjuvant Chemotherapy		
		Yes, n (%)	No, n (%)	*p* Value
Age (years)				0.086
	< 65	68 (71.6)	27 (28.4)	
	≥ 65	43 (84.3)	8 (15.7)	
Gender				0.774
	Female	54 (75.0)	18 (25.0)	
	Male	57 (77.0)	17 (23.0)	
Smoking history				0.360
	No	60 (73.2)	22 (26.8)	
	Yes	51 (79.7)	13 (20.3)	
Nodule				0.770
	Subsolid	5 (71.4)	2 (28.6)	
	Solid	106 (76.3)	33 (23.7)	
CEA (ng/ml)				0.894
	< 5	62 (75.6)	20 (24.4)	
	≥ 5	49 (76.6)	15 (23.4)	
Tumor size (cm)				0.068
	< 3	73 (81.1)	17 (18.9)	
	≥ 3	38 (67.9)	18 (32.1)	
Visceral pleural invasion				0.868
	No	27 (75.0)	9 (25.0)	
	Yes	84 (76.4)	26 (23.6)	
Lymphovascular invasion				0.087
	No	104 (85.2)	18 (14.8)	
	Yes	17 (70.8)	7 (29.2)	
Precedure				0.557
	Sublobectomy	5 (83.3)	1 (16.7)	
	Lobectomy	106 (75.7)	34 (24.3)	
Recurrence				0.021
	No	65 (69.9)	28 (30.1)	
	Yes	46 (86.8)	7 (13.2)	
Lung cancer related death				0.036
	No	79 (71.2)	32 (28.8)	
	Yes	32 (91.4)	3 (8.6)	

**Table 4:** Univariate log-rank test and multivariate Cox regression analysis on prognostic factors related to survival in Group D patients (n=146).

Variable	DFS				OS			
	Univariate	Multivariate			Univariate	Multivariate		
	*p* Value	HR	95% CI	*p*-value	*p*-value	HR	95% CI	*p*-value
Age (≥ 65 vs. < 65)	0.345	1.259	0.700-2.264	0.441	0.064	0.739	0.278-1.960	0.543
Gender (Male vs. Female)	0.915	0.812	0.382-1.726	0.589	0.704	1.699	0.834-3.460	0.144
Smoking history (Yes vs. No)	0.647	0.729	0.336-1.585	0.425	0.588	0.909	0.339-2.443	0.851
CEA (≥ 5 vs. < 5 ng/ml)	0.405	1.312	0.753-2.287	0.337	0.086	1.712	0.860-3.408	0.126
Visceral pleural invasion (Yes vs. No)	0.750	1.630	0.653-4.070	0.295	0.683	1.344	0.474-3.814	0.578
Lymphovascular invasion (Yes vs. No)	0.353	1.193	0.458-3.109	0.717	0.158	0.819	0.191-3.514	0.788
Tumor size (≥ 3 vs. < 3 cm)	0.856	1.781	0.809-3.921	0.152	0.469	1.987	0.779-5.070	0.151
Precedure (Sublobectomy vs. Lobectomy)	0.048	2.735	1.050-7.124	0.039	0.483	1.466	0.415-5.178	0.552
Adjuvant chemotherapy (Yes vs. No)	0.035	0.356	0.135-0.935	0.036	0.046	0.334	0.077-0.852	0.043

## Discussion

In the present study, we discerned a notable correlation between the SUVmax of the primary tumor, as determined through preoperative FDG-PET/CT, and the efficacy of ACT in patients with surgically resected stage I LUAD. An optimal cutoff value for SUVmax (≥ 5) was identified as predictive of postoperative survival following curative resection (**Figure 2**). This threshold, substantiated as an independent prognostic factor in subsequent analyses, holds significant implications, as it delineates a hitherto overlooked demographic poised to benefit from ACT. Our study proposes that patients with pathological stage IB LUAD and SUVmax ≥ 5 represent the bona fide beneficiary cohort for postoperative ACT (**Figure 3**). To the best of our knowledge, our study stands as the first to explore the efficacy of ACT in stage I LUAD, stratified based on the metabolic levels of the primary tumor.

In the context of stage I lung cancers, an estimated 20%–40% are prone to recurrence or metastasis within a five-year period following radical resection, attributed to residual tumor cells that escape surgical elimination (29). Although the consensus supports ACT for patients in high-risk stage I, the controversy surrounding its efficacy in stage I lung cancer persists, leaving uncertainty regarding the specific patient subgroup that stands to benefit (30). The NCCN guidelines prescribe indications for ACT in stage IB NSCLC, including poorly differentiated tumors, vascular invasion, sub-lobar resection, tumors exceeding 4 cm, visceral pleural involvement, and incomplete lymph node sampling (17). However, these risk factors remain unpredictable preoperatively, and not all ostensibly high-risk stage IB patients derive benefit from ACT. Our study reveals that disparities in primary tumor metabolism may exert influence on the efficacy of ACT in stage IB lung cancer. Notably, no discernible survival benefit from ACT was evident in patients with stage IB with lower metabolic levels (SUVmax < 5), even in the presence of these recognized risk factors (**Figure 3**). Conversely, individuals in stage IB with SUVmax ≥ 5 may represent genuine candidates for ACT, and this critical information can be anticipated preoperatively. The discussion regarding the essentiality of postoperative ACT becomes more pronounced for stage IA NSCLCs. Sasada et al. (15) found that ACT had improved the 5-year OS rate (95% vs 81.1%, P = 0.04) in stage IA invasive LUAD after excluding preinvasive lesions and lepidic predominant tumors. Wang et al. (16) conducted a large-scale retrospective study involving 2,633 stage I NSCLC patients and found that ACT was associated with improved survival for stage IA and IB patients with LVI. Other retrospective studies have reported that ACT resulted in a 5-year OS rate improvement of 3% to 5% for stage I lung cancer (13, 14). However, prospective studies, particularly the meta-analysis conducted by the lung adjuvant cisplatin evaluation (LACE) collaboration group, yielded disparate outcomes (13, 14). Although the chemotherapy group exhibited a significantly enhanced prognosis with a 5-year overall survival benefit of 5.4%, ACT failed to enhance survival in patients with stage IA after stratification by stage (HR=1.41, 95% CI: 0.96-2.09). Our study aligns with prospective findings, indicating no survival benefit from ACT in stage IA patients, irrespective of the metabolic level of the primary tumor. Furthermore, in stage IA patients with lower metabolic levels (SUVmax < 5), ACT may lead to a worsened prognosis (**Figure 3**), attributable to potential chemotherapy toxicity.

The susceptibility of tumors to platinum-based chemotherapy may be influenced by the rearrangements of the primary metabolic pathways within cells (25). Chemoresistance and tumor metabolism are intricately linked. SUVmax, one of the most significant indices acquired from PET/CT that can precisely quantify the metabolic activity of tumors, has been used primarily to guide subsequent patient management by means of staging and restaging (31). The European Lung Cancer Working Party determined based a comprehensive review and meta-analysis of 13 studies that tumor SUVmax was a significant predictive factor for NSCLC (32). Increased invasiveness of initial tumors is correlated with a more unfavorable outcome for patients (33). SUVmax ≥ 3.7 for tumors is an independent risk factor for mediastinal lymph node metastasis (34). We discovered in the present study that other prognostic risk variables, including solid nodule, raised CEA level, big tumor size, lymphovascular invasion, and visceral pleural invasion, were substantially linked with an SUVmax ≥ 5 (**Table 1**). Cerfolio et al. discovered in a retrospective study that patients whose SUVmax was ≥ 10 had a higher likelihood of recurrence than those whose SUVmax was relatively low (35). Furthermore, they concluded that SUVmax was a more influential independent prognostic factor than TNM stage. Our research yielded comparable findings: patients diagnosed with stage IA LUAD with an SUVmax > 5 had a poorer DFS and OS in comparison to patients with stage IB with an SUVmax ≥ 5 (**Figure 3**). This suggests that the prognostic influence of tumor SUVmax was more substantial than that of TNM stage.

The primary strength of our study is that, we identified a dependable independent predictor (SUVmax ≥ 5) for DFS and OS among the preoperative risk factors, based on the results of multivariate analysis. Our research findings revealed that patients diagnosed with stage I LUAD who underwent standardized lobectomy and subsequent ACT and had an SUVmax ≥ 5 had a higher risk of lung cancer-related recurrence and death than patients in the lower SUVmax group (as shown in **Table 1**, **Figure 2**). The ideal dichotomous threshold of SUVmax, which is a continuous variable, is crucial for clinical decision-makers to correctly stratify and manage different patient populations. One issue pertains to the variability of SUVmax, a semiquantitative metric that is contingent upon various parameters, including fasting duration, plasma glucose level, time to imaging, reconstruction techniques, and region of interest, which can differ among institutes or PET scanners. Diverse publications have documented SUVmax cutoff values ranging from 2.9 to 5.5 (36–39). The variations in cut-off values among studies can be attributed, in part, to the distinct stages of enrolled cohorts, with the advanced stage being associated with a greater SUVmax (35). Another factor to consider is that distinct tumor histological types have varied metabolic activities.

Prior research has demonstrated that squamous cell carcinomas have more FDG uptake in comparison to adenocarcinomas (39). Furthermore, SUVmax is a more accurate predictor of LUAD prognosis than squamous cell carcinoma (20). For this reason, we concentrated on patients with stage I adenocarcinoma to mitigate the influence of the aforementioned variables (stage and histological type) on the SUVmax cutoff value.

Furthermore, the ROC curve was frequently used in prior research when determining the SUVmax cutoff value. However, the ROC curve is more appropriate for computing variables that have binary outcomes. In contrast, SUVmax exhibits a linear distribution in relation to survival time and is a continuous variable. Therefore, we opted to use the X-tile software to compute the best SUVmax cutoff value instead of other statistical methods for cutoff selection (28, 40, 41). Using the X-tile program and log-rank chi-squared statistics, the ideal SUVmax cut-off values for our study were determined by sifting through many divisions and identifying the minimal p values and maximum hazard ratios in terms of DFS and OS. The cutoff values of SUVmax were 4.9 for DFS and 5.0 for OS, and we chose 5.0 as the threshold because OS is the more widely accepted predictive endpoint.

Our study has several limitations that need to be acknowledged. The first limitation of this study is its retrospective and single-institutional design, which necessitates additional prospective multi-center validation within the context of SUVmax. The second limitation is the unavoidable presence of selection bias. One limitation of our study was the exclusion of pre-invasive and micro-invasive lesions due to the absence of metabolic value on PET/CT and the infrequent occurrence of postoperative recurrence (42). However, considering the potential impact on prognosis, patients diagnosed with LUAD who underwent neoadjuvant therapy or other adjuvant therapies (targeted therapy or radiotherapy) were also excluded. Thirdly, there was no stratification of the study cohort based on molecular profile. Molecular profiling has been demonstrated to be a more accurate predictor of recurrence rates than conventional clinicopathological factors (43, 44). Furthermore, the benefit of ACT for very early stage NSCLC can be predicted for high-risk patients stratified by driver gene mutations (45). The use of targeted treatment as an adjuvant for stage I lung cancer has garnered significant interest in recent times. Based on the subgroup analysis of the ADAURA trial data, adjuvant osimertinib significantly enhanced DFS (HR 0.41) for patients diagnosed with EGFR-mutated stage IB NSCLC compared to placebo (46). Recent retrospective findings indicate that adjuvant EGFR-TKI may increase DFS and OS in patients with NSCLC having stages IA and IB EGFR mutations (47). Unfortunately, only a minor proportion of the patients we had registered had undergone genetic testing. In light of the irregularity observed in the timing and duration of adjuvant targeted therapy, we ultimately made the decision to omit this subset of patients. However, additional research with a more substantial sample size is necessary to compare the effectiveness of ACT and postoperative targeted therapy in patients with early-stage lung cancer.

Despite the limitations outlined, our findings remain valuable, particularly in the context of the non-conflicting nature of ACT and targeted therapy. Our study indicates a lack of benefit from ACT in patients diagnosed with LUAD having stage IA disease and stage IB with low metabolic levels. This observation directs attention toward future investigations that should prioritize exploring the potential advantages of adjuvant targeted therapy. Notably, our results suggest that, for patients in stage IB with high metabolic levels, adjuvant targeted therapy could be beneficial. However, it is crucial to underscore that ACT remains a fundamental treatment, especially for patients with wildtype driver genes. For patients with mutant driver genes, a combined approach of chemotherapy and targeted therapy may represent a viable strategy for adjuvant treatment. We believe that these nuanced considerations merit further scrutiny and validation through prospective clinical studies, allowing for a more comprehensive understanding of their applicability and efficacy in the clinical setting.

## Conclusions

In summary, the preoperative SUVmax of the tumor on FDG-PET/CT emerged as a significant prognostic factor for long-term survival and served as an indicator of the efficacy of ACT in patients with pathologic stage I LUAD following complete resection. Specifically, our findings revealed that among patients diagnosed with stage IB lung adenocarcinoma, those with an SUVmax ≥ 5 constituted the true beneficiaries of postoperative adjuvant chemotherapy. These insights contribute to a deeper comprehension of PET/CT parameters and hold potential implications for the therapeutic decision-making process in the management of patients with LUAD. However, the above conclusions are derived from a retrospective and single-institutional study, and the need for additional large-scale prospective clinical trials is evident to validate the observed survival benefits associated with adjuvant chemotherapy in the context of early-stage lung cancer.

## Data availability statement

The original contributions presented in the study are included in the article/supplementary material. Further inquiries can be directed to the corresponding author.

## Ethics statement

The studies involving humans were approved by Ethical considerations were adhered to in accordance with the Declaration of Helsinki, and approval was obtained from the Ethics Committee of Peking University Cancer Hospital and Institute (Institutional Review Board No. 2019KT59). The studies were conducted in accordance with the local legislation and institutional requirements. The ethics committee/institutional review board waived the requirement of written informed consent for participation from the participants or the participants’ legal guardians/next of kin because Given the retrospective nature of the study, the necessity for informed consent was waived.

## Author contributions

MH: Data curation, Methodology, Project administration, Supervision, Visualization, Writing – original draft, Writing – review & editing. BL: Conceptualization, Data curation, Investigation, Supervision, Writing – review & editing. XL: Formal analysis, Project administration, Validation, Writing – review & editing. NL: Project administration, Resources, Visualization, Writing – review & editing. XY: Data curation, Methodology, Supervision, Writing – review & editing. YW: Conceptualization, Data curation, Investigation, Methodology, Writing – review & editing. SZ: Data curation, Methodology, Project administration, Validation, Writing – review & editing. FL: Formal analysis, Methodology, Project administration, Writing – review & editing. SL: Data curation, Methodology, Project administration, Validation, Writing – review & editing. SY: Data curation, Formal analysis, Methodology, Project administration, Validation, Writing – original draft, Writing – review & editing. NW: Conceptualization, Funding acquisition, Investigation, Methodology, Project administration, Resources, Visualization, Writing – original draft, Writing – review & editing.

## References

[1] Siegel RL Miller KD Fuchs HE Jemal A. Cancer statistics, 2021. CA Cancer J Clin. (2021) 71:7–33. doi: 10.3322/caac.21654 33433946

[2] Chansky K Detterbeck FC Nicholson AG Rusch VW Vallieres E Groome P. The IASLC lung cancer staging project: external validation of the revision of the TNM stage groupings in the eighth edition of the TNM classification of lung cancer. J Thorac Oncol. (2017) 12:1109–21. doi: 10.1016/j.jtho.2017.04.011 28461257

[3] Uramoto H Tanaka F. Recurrence after surgery in patients with NSCLC. Transl Lung Cancer Res. (2014) 3:242–9. doi: 10.3978/j.issn.2218-6751.2013.12.05 PMC4367696 25806307

[4] Sekihara K Hishida T Yoshida J Oki T Omori T Katsumata S. Long-term survival outcome after postoperative recurrence of non-small-cell lung cancer: who is 'cured' from postoperative recurrence? Eur J Cardiothorac Surg. (2017) 52:522–8. doi: 10.1093/ejcts/ezx127 28482033

[5] Sun D Hu J Li X He J Xu L Fu X. Real-world surgical treatment patterns and clinical outcomes in patients with stages IA-IIIA non-small cell lung cancer: a retrospective multicentric observational study involving 11,958 patients. J Cancer Res Clin Oncol. (2023) 149:8213–23. doi: 10.1007/s00432-023-04729-8 PMC11797401 37062036

[6] Arriagada R Bergman B Dunant A Chevalier TL Pignon J-P Vansteenkiste J. Cisplatin-based adjuvant chemotherapy in patients with completely resected non–small-cell lung cancer. N Engl J Med. (2004) 350:351–60. doi: 10.1056/NEJMoa031644 14736927

[7] Winton T Livingston R Johnson D Rigas J Johnston M Butts C. Vinorelbine plus Cisplatin vs. Observation in Resected Non–Small-Cell Lung Cancer. N Engl J Med (2005) 352:2589–97. doi: 10.1056/NEJMoa043623 15972865

[8] Douillard JY Rosell R De Lena M Carpagnano F Ramlau R Gonzales-Larriba JL. Adjuvant vinorelbine plus cisplatin versus observation in patients with completely resected stage IB-IIIA non-small-cell lung cancer (Adjuvant Navelbine International Trialist Association [ANITA]): a randomised controlled trial. Lancet Oncol. (2006) 7:719–27. doi: 10.1016/S1470-2045(06)70804-X 16945766

[9] Pignon JP Tribodet H Scagliotti GV Douillard JY Shepherd FA Stephens RJ. Lung adjuvant cisplatin evaluation: a pooled analysis by the LACE Collaborative Group. J Clin Oncol. (2008) 26:3552–9. doi: 10.1200/JCO.2007.13.9030 18506026

[10] Arriagada R Auperin A Burdett S Higgins JP Johnson DH Le Chevalier T. Adjuvant chemotherapy, with or without postoperative radiotherapy, in operable non-small-cell lung cancer: two meta-analyses of individual patient data. Lancet. (2010) 375:1267–77. doi: 10.1016/S0140-6736(10)60059-1 PMC2853682 20338627

[11] Postmus PE Kerr KM Oudkerk M Senan S Waller DA Vansteenkiste J. Early and locally advanced non-small-cell lung cancer (NSCLC): ESMO Clinical Practice Guidelines for diagnosis, treatment and follow-up. Ann Oncol. (2017) 28:iv1–iv21. doi: 10.1093/annonc/mdx222 28881918

[12] Kris MG Gaspar LE Chaft JE Kennedy EB Azzoli CG Ellis PM. Adjuvant systemic therapy and adjuvant radiation therapy for stage I to IIIA completely resected non–small-cell lung cancers: american society of clinical oncology/cancer care ontario clinical practice guideline update. J Clin Oncol. (2017) 35:2960–74. doi: 10.1200/JCO.2017.72.4401 28437162

[13] Kato H Ichinose Y Ohta M Hata E Tsubota N Tada H. A randomized trial of adjuvant chemotherapy with uracil–tegafur for adenocarcinoma of the lung. N Engl J Med. (2004) 350:1713–21. doi: 10.1056/NEJMoa032792 15102997

[14] Hamada C Tanaka F Ohta M Fujimura S Kodama K Imaizumi M. Meta-analysis of postoperative adjuvant chemotherapy with tegafur-uracil in non-small-cell lung cancer. J Clin Oncol. (2005) 23:4999–5006. doi: 10.1200/JCO.2005.09.017 16051951

[15] Sasada S Miyata Y Mimae T Mimura T Okada M. Impact of lepidic component occupancy on effects of adjuvant chemotherapy for lung adenocarcinoma. Ann Thorac Surg. (2015) 100:2079–86. doi: 10.1016/j.athoracsur.2015.05.102 26298165

[16] Wang S Xu J Wang R Qian F Yang W Qiao R. Adjuvant chemotherapy may improve prognosis after resection of stage I lung cancer with lymphovascular invasion. J Thorac Cardiovasc Surg. (2018) 156:2006–15.e2002. doi: 10.1016/j.jtcvs.2018.06.034 30104070

[17] Ettinger DS Wood DE Aisner DL Akerley W Bauman JR Bharat A. Non-small cell lung cancer, version 3.2022, NCCN clinical practice guidelines in oncology. J Natl Compr Canc Netw. (2022) 20:497–530. doi: 10.6004/jnccn.2022.0025 35545176

[18] Warth A Muley T Meister M Stenzinger A Thomas M Schirmacher P. The novel histologic International Association for the Study of Lung Cancer/American Thoracic Society/European Respiratory Society classification system of lung adenocarcinoma is a stage-independent predictor of survival. J Clin Oncol. (2012) 30:1438–46. doi: 10.1200/JCO.2011.37.2185 22393100

[19] Cooper WA Lam DC O'Toole SA Minna JD. Molecular biology of lung cancer. J Thorac Dis. (2013) 5 Suppl 5:S479–490. doi: 10.3978/j.issn.2072-1439.2013.08.03 PMC3804875 24163741

[20] Tsutani Y Miyata Y Misumi K Ikeda T Mimura T Hihara J. Difference in prognostic significance of maximum standardized uptake value on [18F]-fluoro-2-deoxyglucose positron emission tomography between adenocarcinoma and squamous cell carcinoma of the lung. Jpn J Clin Oncol. (2011) 41:890–6. doi: 10.1093/jjco/hyr062 21613306

[21] Tsutani Y Miyata Y Nakayama H Okumura S Adachi S Yoshimura M. Solid tumor size on high-resolution computed tomography and maximum standardized uptake on positron emission tomography for new clinical T descriptors with T1 lung adenocarcinoma. Ann Oncol. (2013) 24:2376–81. doi: 10.1093/annonc/mdt230 23788749

[22] Karam MB Doroudinia A Behzadi B Mehrian P Koma AY. Correlation of quantified metabolic activity in nonsmall cell lung cancer with tumor size and tumor pathological characteristics. Med (Baltimore). (2018) 97:e11628. doi: 10.1097/MD.0000000000011628 PMC6133455 30095621

[23] Carretta A Bandiera A Muriana P Viscardi S Ciriaco P Gajate AMS. Prognostic role of positron emission tomography and computed tomography parameters in stage I lung adenocarcinoma. Radiol Oncol. (2020) 54:278–84. doi: 10.2478/raon-2020-0034 PMC7409601 32463388

[24] Williams CD Gajra A Ganti AK Kelley MJ. Use and impact of adjuvant chemotherapy in patients with resected non-small cell lung cancer. Cancer. (2014) 120:1939–47. doi: 10.1002/cncr.28679 24668613

[25] Cocetta V Ragazzi E Montopoli M. Links between cancer metabolism and cisplatin resistance. Int Rev Cell Mol Biol. (2020) 354:107–64. doi: 10.1016/bs.ircmb.2020.01.005 32475471

[26] Strauss GM Herndon JE 2nd Maddaus MA Johnstone DW Johnson EA Harpole DH. Adjuvant paclitaxel plus carboplatin compared with observation in stage IB non-small-cell lung cancer: CALGB 9633 with the Cancer and Leukemia Group B, Radiation Therapy Oncology Group, and North Central Cancer Treatment Group Study Groups. J Clin Oncol. (2008) 26:5043–51. doi: 10.1200/JCO.2008.16.4855 PMC2652093 18809614

[27] Szejniuk WM Cekala M Bogsted M Meristoudis C McCulloch T Falkmer UG. Adjuvant platinum-based chemotherapy in non-small cell lung cancer: The role of relative dose-intensity and treatment delay. Cancer Treat Res Commun. (2021) 27:100318. doi: 10.1016/j.ctarc.2021.100318 33515937

[28] Camp RL Dolled-Filhart M Rimm DL. X-tile: A new bio-informatics tool for biomarker assessment and outcome-based cut-point optimization. Clin Cancer Res. (2004) 10:7252–9. doi: 10.1158/1078-0432.CCR-04-0713 15534099

[29] Goldstraw P Chansky K Crowley J Rami-Porta R Asamura H Eberhardt WE. The IASLC lung cancer staging project: proposals for revision of the TNM stage groupings in the forthcoming (Eighth) edition of the TNM classification for lung cancer. J Thorac Oncol. (2016) 11:39–51. doi: 10.1016/j.jtho.2015.09.009 26762738

[30] Park JH Lee CT Lee HW Baek HJ Zo JI Shim YM. Postoperative adjuvant chemotherapy for stage I non-small cell lung cancer. Eur J Cardiothorac Surg. (2005) 27:1086–91. doi: 10.1016/j.ejcts.2005.01.039 15896623

[31] Wang Y Ma S Dong M Yao Y Liu K Zhou J. Evaluation of the factors affecting the maximum standardized uptake value of metastatic lymph nodes in different histological types of non-small cell lung cancer on PET-CT. BMC Pulm Med. (2015) 15:20. doi: 10.1186/s12890-015-0014-2 25880540 PMC4372315

[32] Berghmans T Dusart M Paesmans M Hossein-Foucher C Buvat I Castaigne C. Primary Tumor Standardized Uptake Value (SUVmax) Measured on Fluorodeoxyglucose Positron Emission Tomography (FDG-PET) is of Prognostic Value for Survival in Non-small Cell Lung Cancer (NSCLC): a systematic review and meta-analysis (MA) by the European Lung Cancer Working Party for the IASLC Lung Cancer Staging Project. J Thorac Oncol. (2008) 3:6–12. doi: 10.1097/JTO.0b013e31815e6d6b 18166834

[33] Hattori A Matsunaga T Takamochi K Oh S Suzuki K. Clinical significance of positron emission tomography in subcentimeter non-small cell lung cancer. Ann Thorac Surg. (2017) 103:1614–20. doi: 10.1016/j.athoracsur.2016.09.059 27964917

[34] Miao H Shaolei L Nan L Yumei L Shanyuan Z Fangliang L. Occult mediastinal lymph node metastasis in FDG-PET/CT node-negative lung adenocarcinoma patients: Risk factors and histopathological study. Thorac Cancer. (2019) 10:1453–60. doi: 10.1111/1759-7714.13093 PMC6558456 31127706

[35] Cerfolio RJ Bryant AS Ohja B Bartolucci AA. The maximum standardized uptake values on positron emission tomography of a non-small cell lung cancer predict stage, recurrence, and survival. J Thorac Cardiovasc Surg. (2005) 130:151–9. doi: 10.1016/j.jtcvs.2004.11.007 15999056

[36] Ohtsuka T Nomori H Watanabe K Kaji M Naruke T Suemasu K. Prognostic significance of [(18)F]fluorodeoxyglucose uptake on positron emission tomography in patients with pathologic stage I lung adenocarcinoma. Cancer. (2006) 107:2468–73. doi: 10.1002/cncr.22268 17036361

[37] Shiono S Abiko M Sato T. Positron emission tomography/computed tomography and lymphovascular invasion predict recurrence in stage I lung cancers. J Thorac Oncol. (2011) 6:43–7. doi: 10.1097/JTO.0b013e3181f9abca 21079522

[38] Uehara H Tsutani Y Okumura S Nakayama H Adachi S Yoshimura M. Prognostic role of positron emission tomography and high-resolution computed tomography in clinical stage IA lung adenocarcinoma. Ann Thorac Surg. (2013) 96:1958–65. doi: 10.1016/j.athoracsur.2013.06.086 24021765

[39] Goodgame B Pillot GA Yang Z Shriki J Meyers BF Zoole J. Prognostic value of preoperative positron emission tomography in resected stage I non-small cell lung cancer. J Thorac Oncol. (2008) 3:130–4. doi: 10.1097/JTO.0b013e318160c122 18303432

[40] Mazumdar M Glassman JR. Categorizing a prognostic variable: review of methods, code for easy implementation and applications to decision-making about cancer treatments. Stat Med. (2000) 19:113–32. doi: 10.1002/(sici)1097-0258(20000115)19:1<113::aid-sim245>3.0.co;2-o 10623917

[41] Mazumdar M Smith A Bacik J. Methods for categorizing a prognostic variable in a multivariable setting. Stat Med. (2003) 22:559–71. doi: 10.1002/sim.1333 12590414

[42] Tsao MS Nicholson AG Maleszewski JJ Marx A Travis WD. Introduction to 2021 WHO classification of thoracic tumors. J Thorac Oncol. (2022) 17:e1–4. doi: 10.1016/j.jtho.2021.09.017 34930611

[43] Kratz JR He J Van Den Eeden SK Zhu ZH Gao W Pham PT. A practical molecular assay to predict survival in resected non-squamous, non-small-cell lung cancer: development and international validation studies. Lancet. (2012) 379:823–32. doi: 10.1016/S0140-6736(11)61941-7 PMC3294002 22285053

[44] Woodard GA Gubens MA Jahan TM Jones KD Kukreja J Theodore PR. Prognostic molecular assay might improve identification of patients at risk for recurrence in early-stage non-small-cell lung cancer. Clin Lung Cancer. (2014) 15:426–32. doi: 10.1016/j.cllc.2014.07.004 25258195

[45] Woodard GA Wang SX Kratz JR Zoon-Besselink CT Chiang CY Gubens MA. Adjuvant chemotherapy guided by molecular profiling and improved outcomes in early stage, non-small-cell lung cancer. Clin Lung Cancer. (2018) 19:58–64. doi: 10.1016/j.cllc.2017.05.015 28645632

[46] Herbst RS Wu Y-L John T Grohe C Majem M Wang J. Adjuvant osimertinib for resected EGFR-mutated stage IB-IIIA non-small-cell lung cancer: updated results from the phase III randomized ADAURA trial. J Clin Oncol. (2023) 41:1830–40. doi: 10.1200/JCO.22.02186 PMC10082285 36720083

[47] Jiang Y Lin Y Fu W He Q Liang H Zhong R. The impact of adjuvant EGFR-TKIs and 14-gene molecular assay on stage I non-small cell lung cancer with sensitive EGFR mutations. EClinicalMedicine. (2023) 64:102205. doi: 10.1016/j.eclinm.2023.102205 37745018 PMC10511786

